# Clinical Low Dose Photon Counting CT for the Detection of Urolithiasis: Evaluation of Image Quality and Radiation Dose

**DOI:** 10.3390/tomography8040138

**Published:** 2022-06-23

**Authors:** Julius Henning Niehoff, Alexandra Fiona Carmichael, Matthias Michael Woeltjen, Jan Boriesosdick, Ingo Lopez Schmidt, Arwed Elias Michael, Nils Große Hokamp, Hansjuergen Piechota, Jan Borggrefe, Jan Robert Kroeger

**Affiliations:** 1 Department of Radiology, Neuroradiology and Nuclear Medicine, Johannes Wesling University Hospital, Ruhr University Bochum, 44801 Bochum, Germany; matthiasmichael.woeltjen@muehlenkreiskliniken.de (M.M.W.); jan.boriesosdick@muehlenkreiskliniken.de (J.B.); ingo.lopezschmidt@muehlenkreiskliniken.de (I.L.S.); arwed.michael@muehlenkreiskliniken.de (A.E.M.); jan.borggrefe@muehlenkreiskliniken.de (J.B.); janrobert.kroeger@muehlenkreiskliniken.de (J.R.K.); 2 Department of Urology, Johannes Wesling University Hospital, Ruhr University Bochum, 44801 Bochum, Germany; alexandrafiona.carmichael@muehlenkreiskliniken.de (A.F.C.); hansjuergen.piechota@muehlenkreiskliniken.de (H.P.); 3 Faculty of Medicine and University Hospital Cologne, Institute for Diagnostic and Interventional Radiology, University of Cologne, 50937 Cologne, Germany; nils.grosse-hokamp@uk-koeln.de

**Keywords:** photon counting CT, low-dose CT, CT image quality, CT radiation dose, urolithiasis

## Abstract

The purpose of this study was the evaluation of image quality and radiation dose parameters of the novel photon counting CT (PCCT, Naeotom Alpha, Siemens Healthineers) using low-dose scan protocols for the detection of urolithiasis. Standard CT scans were used as a reference (S40, Somatom Sensation 40, Siemens Healthineers). Sixty-three patients, who underwent CT scans between August and December 2021, were retrospectively enrolled. Thirty-one patients were examined with the PCCT and 32 patients were examined with the S40. Radiation dose parameters, as well as quantitative and qualitative image parameters, were analyzed. The presence of urolithiasis, image quality, and diagnostic certainty were rated on a 5-point-scale by 3 blinded readers. Both patient groups (PCCT and S40) did not differ significantly in terms of body mass index. Radiation dose was significantly lower for examinations with the PCCT compared to the S40 (2.4 ± 1.0 mSv vs. 3.4 ± 1.0 mSv; *p* < 0.001). The SNR was significantly better on images acquired with the PCCT (13.3 ± 3.3 vs. 8.2 ± 1.9; *p* < 0.001). The image quality of the PCCT was rated significantly better (4.3 ± 0.7 vs. 2.8 ± 0.6; *p* < 0.001). The detection rate of kidney or ureter calculi was excellent with both CT scanners (PCCT 97.8% and S40 99%, *p* = 0.611). In high contrast imaging, such as the depiction of stones of the kidney and the ureter, PCCT allows a significant reduction of radiation dose, while maintaining excellent diagnostic confidence and image quality. Given this image quality with our current protocol, further adjustments towards ultra-low-dose CT scans appear feasible.

## 1. Introduction

Stones of the kidney and ureter that cause renal colic pain are a very common reason for hospital referral. In the United States (US), the overall lifetime prevalence of urolithiasis in adults has increased from 3.2% in 1976–1980 to 8.8% in 2007–2010, and the overall incidence increased from 0.6% to 0.9% between 2005 and 2015 [1,2,3].

The highest prevalence of urolithiasis is observed in elderly patients. However, the occurrence is not limited to older generations—kidney and ureter stones can also be developed by young patients [2]. It has been shown in various studies that the recurrence rate after a first symptomatic episode of urolithiasis is considerably high [1,4]. Consequently, as patients admitted to the hospital with renal colic can be of young age and will potentially experience more than one episode of urolithiasis in their lifetime, the appropriate diagnostic technique must be chosen carefully.

Although modern medicine offers different imaging techniques for the detection of urinary calculi, there is a general consensus that non-contrast CT scans offer the highest sensitivity and specificity for the detection of kidney and ureter stones [5]. Alternative imaging techniques, such as MRI, ultrasonography, or plain film radiography, provide lower sensitivity and specificity [5,6].

However, CT examinations always involve exposure to ionizing radiation. Therefore, strategies to reduce radiation dose for the patient are of great interest. Various low-dose CT scan protocols pursuing different approaches have been developed in the past. The reduction of radiation dose is often achieved by reducing the tube charge current [7]. Other approaches, among many others, are based on an increased pitch factor, an additional tin filtration or iterative reconstructions [8,9,10,11,12,13,14].

The definition of “low-dose” or “ultra-low-dose” CT scan protocols for the detection of urolithiasis varies slightly in the literature. Rob et al., for example, reporting on a systematic review of different scan protocols, defined radiation doses of ≤3.5 mSv as “low-dose” and ≤1.9 mSv as “ultra-low-dose” [15]. Weisenthal et al., also conducting a systematic review, defined the term “reduced-dose CT” as a CT scan with a dose length product (DLP) of 200 mGy*cm or less [16].

In 2021, a new approach to reduce radiation exposure was introduced with the launch of the first photon counting CT (PCCT) approved for clinical use. Unlike conventional energy-integrating detectors (EID) that have been used in the past, the photon counting detector (PCD) consists of a single thick layer of a semiconductor material that converts x-rays directly into an electrical signal [17,18]. Prior to the introduction of the first clinical PCCT, photon counting technology has been used for clinical purposes in the field of mammography with great success [19,20,21].

Based on early studies conducted with CT prototypes, PCDs are believed to offer several advantages in comparison to EIDs, e.g., a reduction of image noise and an improvement of the contrast to noise ratio. Furthermore, PCDs can potentially lead to a significant reduction in radiation dose exposure [17]. Kappler et al. report on a radiation dose reduction of up to 32% with increased (iodine) contrast and similar image noise compared to EIDs [17,22]. Recently published studies reporting on the general performance of the first PCCT that is approved for clinical use describe promising results [23,24,25,26,27]. However, the potential of the novel PCCT to reduce the radiation dose in the clinical routine has not yet been evaluated in detail.

The purpose of the present study is to evaluate the image quality and the radiation dose parameters of the PCCT using a low-dose scan protocol for the detection of urolithiasis. Furthermore, we compare the image quality as well as the radiation dose of the PCCT with a standard CT scanner.

## 2. Materials and Methods

### 2.1. Patient Population

The study was conducted according to the guidelines of the Declaration of Helsinki and approved by the institutional review board. Patient consent was waived due to the retrospective study design.

All scans were performed for diagnostic reasons with the clinical suspicion of urolithiasis. A total of 63 consecutive patients, who underwent a CT scan using either the PCCT or the standard CT between August and December 2021, were retrospectively enrolled in this study. Patients were not preselected regarding weight, age, gender, or other characteristics. Patient data were pseudonymized.

### 2.2. CT Protocols and Image Acquisition

All CT examinations were performed in a supine position. Standard scan protocols were used for the CT examinations with both CT scanners.

Scan parameters for all examinations with the PCCT (Naeotom Alpha, software version Syngo CT VA40, Siemens Healthineers, Erlangen, Germany) were as follows: tube voltage 100 kV, detector configuration 144 × 0.4 mm, automatic tube current modulation, image quality (IQ) level 70, tin filter, pitch 0.6, gantry rotation time 0.5 s. The reconstruction parameters were as follows: slice thickness 2 mm, image matrix 512 × 512, soft tissue (body) kernel, and quantum iterative reconstruction (QIR) level Q4.

Scan parameters for all examinations with the standard CT scanner (Somatom Sensation 40 (S40), Siemens Healthineers, Erlangen, Germany) were as follows: tube voltage 120 kVp, detector configuration 24 × 1.2 mm, automatic tube current modulation, reference mAs 90, pitch 1, gantry rotation time 0.5 s. The reconstruction parameters were as follows: slice thickness 3 mm, image matrix 512 × 512, and soft tissue (body) kernel.

### 2.3. Radiation Dose

The computed tomography dose index (CTDI), the dose length product (DLP), and the effective dose (ED) were analyzed. The ED was calculated as explained by Stamm et al. [28]. Subgroups were formed with regard to gender and body mass index (BMI). In addition, the size-specific dose estimates (SSDE) were analyzed.

### 2.4. Quantitative Image Analysis

Regions of interest (ROI) were drawn in the subcutaneous fat and the paravertebral muscle. The ROIs were constant in size (diameter 1 cm) on all images. The mean density and the standard deviation (SD) of each ROI were recorded. The SD of each ROI was defined as image noise. The signal-to-noise ratio (SNR) was calculated (mean HU divided by the SD of each ROI).

### 2.5. Qualitative Image Analysis

Three radiologists with 19 years, 3 years, and 2 years of experience evaluated the CT images independently in terms of overall image quality, image noise, and image sharpness on a 5-point Likert scale (see Table 1). Furthermore, the radiologists declared whether they detected a kidney or ureter stone on each particular CT scan and rated how confident they felt with their diagnosis on a 5-point Likert scale (5 = very confident, 1 = not confident). The CT scans were evaluated by the readers in random order using the established IMPAX system (version 6.7, Agfa Healthcare, Mortsel, Belgium). Prior to the reading, all patient-identifying information was replaced and the information about the CT scanner was hidden from the CT images. Ground truth was based on the radiology report and expert consensus reading in discordant cases.

### 2.6. Statistical Analysis

Established software packages were used for the statistical analysis (SPSS Statistics 28, IBM, Armonk, NY, USA; Excel 2016, Microsoft, Redmond, WA, USA; R Core Team (2021). R: A language and environment for statistical computing. R Foundation for Statistical Computing, Vienna, Austria; RStudio Version 1.4.1106). If not stated otherwise, all data are presented as mean ± standard deviation (SD). For the qualitative reading, differences of the mean between both CT scanners were calculated using the Mann–Whitney U test. Results are visualized as horizontal stacked bar charts. For radiation dose variables, BMI and SNR, differences in the mean were calculated using an independent two-sample *t*-test as normal distribution was assumed based on histogram analysis and sample size. However, because of the reduced sample size in the subgroup analysis of these parameters, the more robust non-parametric testing (Mann–Whitney U test) was employed. Differences are visualized as box plots. For the detection rate, differences between both CT scanners were tested using the Fisher’s exact test. A *p*-value of less than 0.05 was considered indicative of statistical significance.

## 3. Results

### 3.1. Patient Population

**Figure 1:**
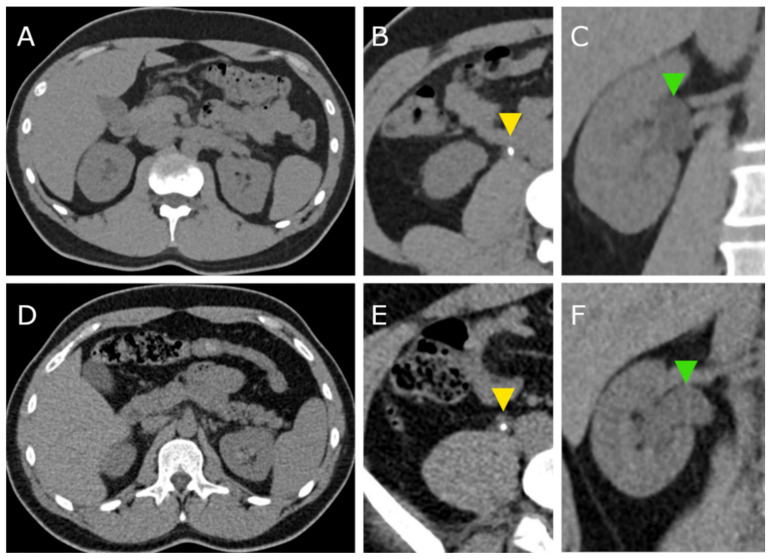
CT images acquired with the photon counting CT (PCCT, **A**–**C**) and with the standard CT (S40, **D**–**E**). Yellow arrowheads (**B**,**E**) indicate ureter stones in both patients. Green arrowheads (**C**,**F**) point to the dilated renal pelvis due to the ureter obstruction.

In total, CT scans of 63 patients were included in the analysis. Thirty-one patients (16 female, 15 male) were examined with the PCCT and 32 patients (11 female, 21 male) were examined with the S40. Figure 1 exemplarily shows CT images of both scanners.

The mean age of all patients examined with the PCCT was 51 years (range 21–94 years). The mean age of all patients examined with the S40 was 46 years (range 18–68 years). There was no significant difference between the patients examined with the PCCT and the S40 in terms of body mass indices (BMI, see also Table 2).

### 3.2. Radiation Dose

**Figure 2:**
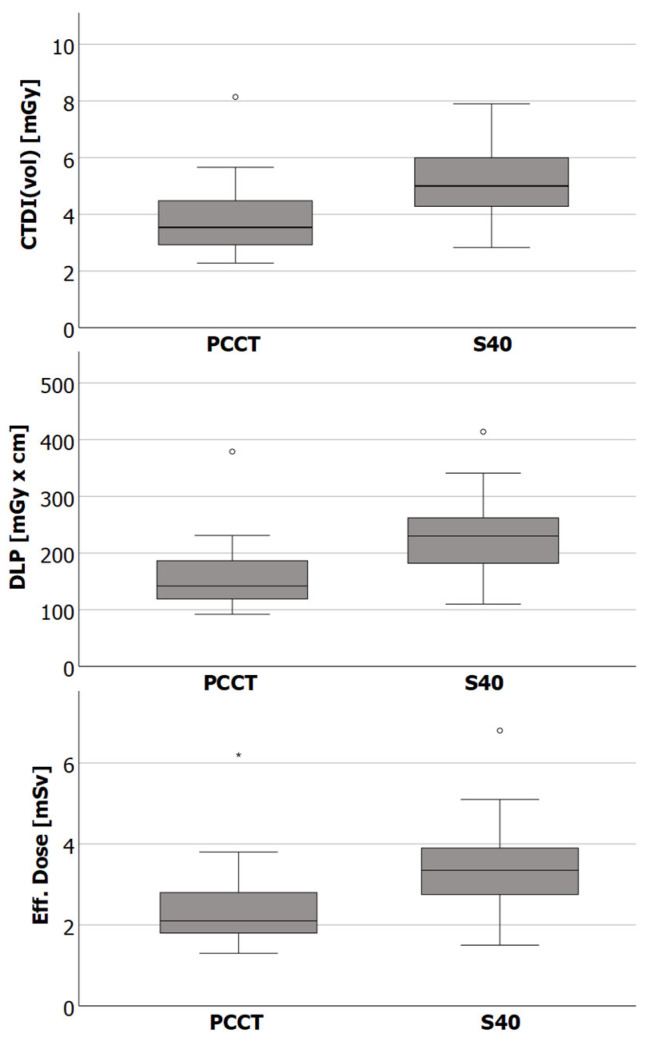
Box plots showing the median, the lower (Q1), and upper (Q3) quartile, as well as the interquartile range * 1.5 (whiskers) of the computed tomography dose index (CTDIvol), the dose length product (DLP) and the effective dose of CT examinations with the photon counting CT (PCCT) and the standard CT (S40). Differences between CT scanners were statistically significant for all dose indices.

CTDI_vol_, DLP, and ED are presented in Table 3 and visualized in Figure 2. Subgroups were formed with regard to gender and BMI. Overall, the ED as well as the SSDE was significantly lower for examinations with the PCCT compared to examinations with the S40 (ED 2.4 ± 1.0 mSv vs. 3.4 ± 1.0 mSv; *p* < 0.001; SSDE 4.6 ± 0.9 mGy vs. 5.8 ± 1.1 mGy, *p* < 0.001). The PCCT also required significantly less radiation dose when considering each subgroup. The difference in radiation dose was greatest among male patients (ED 2.0 ± 0.5 mSv vs. 3.3 ± 0.8 mSv, *p* < 0.001).

### 3.3. Quantitative Image Analysis

The SNR was significantly higher on images acquired with the PCCT compared to the SNR on images acquired with the S40. This applies to the subcutaneous fat (SNR 13.3 ± 3.3 vs. 8.2 ± 1.9; *p* < 0.001) as well as to muscle (SNR 6.2 ± 1.5 vs. 2.5 ± 0.6; < 0.001). At the same time, the image noise was significantly lower on images acquired with the PCCT compared to the S40, which also applies to measurements in subcutaneous fat (8.6 ± 2.3 vs. 14.8 ± 3.0; *p* < 0.001) and muscle (9.1 ± 2.0 vs. 18.8 ± 3.2; *p* < 0.001).

### 3.4. Qualitative Image Analysis

Taking all CT examinations with the PCCT into account, there was one false negative and one false positive diagnosis among all three radiologists, leading to a detection rate of 97.8% for urolithiasis. Taking all CT examinations with the S40 into account, there was only one false negative diagnosis among all three radiologists, leading to a detection rate of 99%. The detection rates of both CT scanners did not differ significantly (*p* = 0.611).

**Figure 3:**
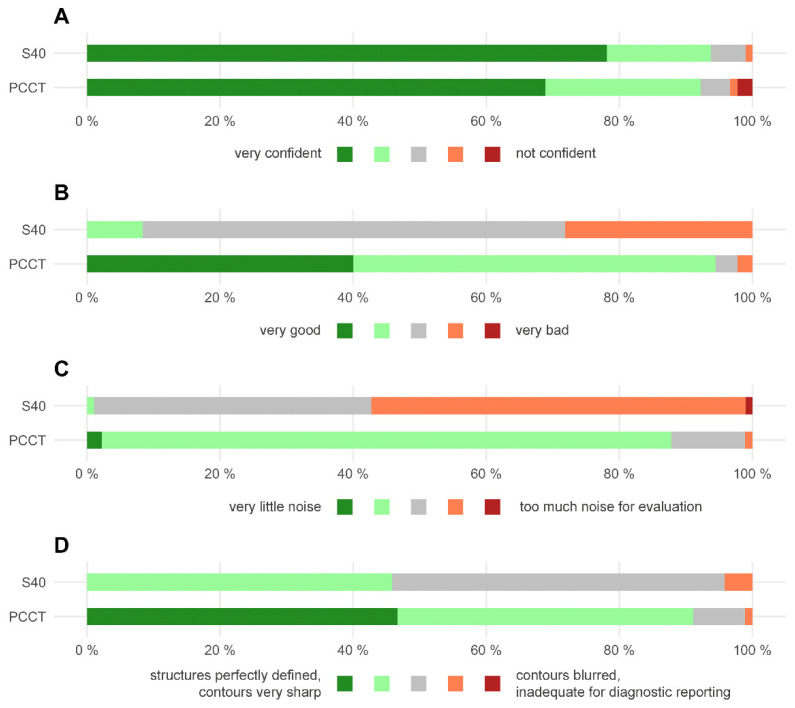
Horizontal stacked bar chart displaying the qualitative image analysis. (**A**) Diagnostic confidence. (**B**) overall image quality. (**C**) image noise. (**D**) image sharpness. PCCT = photon counting CT, S40 = standard CT.

The diagnostic confidence was high for examinations with both CT scanners and did not differ significantly (4.6 ± 0.8 (PCCT) vs. 4.7 ± 0.6 (S40); *p* = 0.162; see also Figure 3). The overall image quality was rated significantly higher for CT examination with the PCCT (4.3 ± 0.7 vs. 2.8 ± 0.6; *p* < 0.001). Likewise, the image noise and the image sharpness were rated significantly better for scans acquired with the PCCT (image noise 3.9 ± 0.4 vs. 2.4 ± 0.5; *p* < 0.001 and image sharpness 4.4 ± 0.7 vs. 3.4 ± 0.6; *p* < 0.001).

## 4. Discussion

The subject of the present study was the assessment of the image quality and the radiation dose parameters of the PCCT using a low-dose scan protocol for the detection of urolithiasis. Furthermore, a comparison was made with a standard CT scanner.

The comparison of the radiation dose parameters of both CT scanners in the present study shows that examinations with the PCCT required a significantly lower radiation dose. However, this comparison is hampered by the differing scan protocols of both scanners. Nevertheless, the radiation dose parameters of the PCCT also fit very well into the context of the literature. Rob et al., for example, considered scan protocols for the detection of urolithiasis that utilized radiation doses of ≤3.5 mSv as “low-dose” [15]. The mean radiation dose among all patients utilized by the PCCT (2.4 mSv) in the present study is far below the limit of this definition of “low-dose”. In fact, the mean radiation dose among male patients (2.0 mSv) is only slightly above the limit of an “ultra-low-dose” scan protocol (≤1.9 mSv) as defined by Rob et al. [15]. With a mean DLP of 158.2 mGy*cm among all patients, the PCCT also performs very well in terms of radiation dose when considering the definition of a “reduced-dose CT” (DLP ≤ 200 mGy*cm) set by Weisenthal et al. [16].

Recently published studies based on modern dual-energy CT scanners with optimized scan protocols for the detection and evaluation of kidney and ureter stones report on effective doses of slightly over 3 mSv [10,14,29,30,31]. Although a direct comparison of these studies with the present study appears inappropriate, it nevertheless points to the great potential of PCD detectors to reduce the radiation dose for patients. As the overall image quality, as well as the image noise and sharpness, were rated as more than sufficient in this study, it even seems feasible to further reduce the radiation dose by adjusting the PCCT scan protocol.

In this context, the study conducted by Decker et al. seems interesting [23]. They also performed low-dose CT scans of the abdomen with a PCCT. Different from the present study, they lowered the image quality level in their scan protocol to 25 (compared to an image quality level of 70 in the present study). They report a mean DLP of 63.5 ± 19.6 mGy*cm and an SNR of 2.31 ± 0.3 in the psoas muscle (compared to an SNR of 6.2 ± 1.5 in the present study) [23]. Therefore, the study of Decker et al. provides an additional indication that the radiation dose can be further reduced in principle. Nevertheless, it seems reasonable to reduce the radiation dose stepwise in order to ensure that diagnostic accuracy is not compromised.

In the present study, the overall image quality, as well as the image noise and sharpness of CT examinations with the PCCT, were rated significantly better compared to examinations with the standard CT scanner by all three radiologists. This seems particularly interesting since the slightly thicker slice thickness (3 mm versus 2 mm) used by the standard CT scanner should be an advantage in terms of subjectively perceived image noise. The subjective perception of the superior image quality of the PCCT was confirmed by the quantitative image analysis. The images acquired with the PCCT offer a significantly higher SNR compared to the images acquired with the S40—this applies to measurements in the subcutaneous fat as well as in muscle. These are highly interesting findings as the PCCT used a significantly lower radiation dose compared to the standard CT scanner, which is usually associated with lower image quality. Despite the lower image quality of the S40, the detection rate for urolithiasis was excellent with both scanners and did not differ significantly. The detection rate in the present study is within the range that has been reported in previous studies [32]. There was no significant difference in diagnostic confidence of the radiologists between both CT scanners.

The present study has certain limitations. As mentioned above, CT examinations with the PCCT required a significantly lower radiation dose compared to CT examinations with the S40. However, differences in radiation doses between both CT scanners must be assessed with caution as the scan protocols differed between both scanners. For example, the potential influence of the different pitches on radiation doses was not taken into account in this comparison. An adjustment of the scan parameters for the scan protocol of the S40, which may have led to a reduction in the radiation dose, would inevitably have been accompanied by a deterioration of the image quality, which was already inferior to the PCCT with the given scan protocol. Thus, we are not able to assess the influence of individual aspects of the scanner technologies that may have influenced radiation dose and image quality, e.g., tin filter, iterative reconstruction, and detector technology. A direct comparison of the PCCT to a latest generation CT scanner with EID would be preferable in order to describe the improvements based on detector technology in more detail. However, no such scanner is available at our institute. Furthermore, the qualitative analysis was focused on image quality criteria. The size and composition of the calculi, as well as the influence of the image quality on the measurement, was not addressed.

## 5. Conclusions

In conclusion, the PCCT performed very well in the imaging of stones in the kidney and ureter. The image quality offered by the PCCT led to a high diagnostic confidence and excellent detection rate of urolithiasis. At our radiological institute, the current PCCT scan protocol for the detection of urolithiasis uses significantly less radiation dose (about 30%) compared to our previous standard CT scanner. The results of the present study allow further adjustments to our clinical scan protocol in order to reduce the radiation dose for our patients.

## Figures and Tables

**Table 1:** Criteria for the evaluation of the image quality (adapted from Mozaffary et al., 2019).

Score	Overall Image Quality	Image Noise	Image Sharpness
**5**	Very Good	Very little noise	Structures are **perfectly defined,** Contours are **very sharp**
**4**	Good	Appropriate noise	Structures are **well defined,** Contours are **sharp**
**3**	Sufficient	Noisy, evaluation is possible	Structures are **defined**, Contours are **not fully sharp**
**2**	Bad	Very noisy, evaluation is difficult	Structures can be seen, Contours **are barely sharp** enough
**1**	Very bad	Too much noise for evaluation	Contours **are blurred,** images are **inadequate for diagnostic reporting**

**Table 2:** Body mass indices (BMI). Mean ± SD.

	n	BMI (kg/m^2^)			
	PCCT	S40	PCCT	S40	*p*
**Total**	31	32	**27.5** ± 5.1	**29.0** ± 5.4	0.268
**Total, BMI < 30**	23	21	**25.1** ± 3.2	**26.0** ± 2.8	0.353
**Male**	15	21	**27.4** ± 4.5	**29.5** ± 4.3	0.191
**Female**	16	11	**27.6** ± 5.7	**28.0** ± 7.2	0.981

**Table 3:** Computed tomography dose index (CTDIvol), dose length product (DLP), and effective dose of all patients examined with the photon counting CT (PCCT) and with the standard CT (S40). Mean ± SD.

	CTDI_vol_ (mGy)			DLP (mGy*cm)			Eff. Dose (mSv)		
	PCCT	S40	*p*	PCCT	S40	*p*	PCCT	S40	*p*
**Total**	**3.8** ± 1.2	**5.2** ± 1.5	<0.001	**158.2** ± 58.0	**232.5** ± 67.6	<0.001	**2.4** ± 1.0	**3.4** ± 1.0	<0.001
**Total,** **BMI < 30**	**3.4** ± 0.8	**4.5** ± 1.1	<0.001	**138.3** ± 33.7	**203.9** ± 62.5	<0.001	**2.1** ± 0.6	**3.1** ± 1.0	<0.001
**Male**	**3.5** ± 0.7	**5.1** ± 1.3	<0.001	**144.7** ± 37.8	**231.2** ± 55.8	<0.001	**2.0** ± 0.5	**3.3** ± 0.8	<0.001
**Female**	**4.1** ± 1.5	**5.3** ± 1.8	0.080	**170.8** ± 71.0	**235.0** ± 89.0	0.030	**2.8** ± 1.2	**3.8** ± 1.4	0.034

## Data Availability

The data are available from the corresponding author on reasonable request.
